# Losing Sleep, Gaining Weight? Young Bangladeshi Adults' Perceptions of Sleep, Eating and Body Weight: A Qualitative Study

**DOI:** 10.1111/hex.70933

**Published:** 2026-10-16

**Authors:** Mst Sabrina Moonajilin, Rajib Ul Islam, Labina Taher, Fariha Noshin, Tabeen Taher, Sabkat Kamal

**Affiliations:** ^1^ Department of Public Health and Informatics Jahangirnagar University Savar Dhaka Bangladesh; ^2^ Psychology and Counselling, Queensland University of Technology Brisbane Queensland Australia; ^3^ Department of Government & Politics Jahangirnagar University Savar Dhaka Bangladesh; ^4^ Kelvin Grove State College Brisbane Queensland Australia; ^5^ Govt. College of Applied Human Science Dhaka Bangladesh; ^6^ Department of Urban & Regional Planning Jahangirnagar University Savar Dhaka Bangladesh

**Keywords:** excess weight gain, sleep quality, socio‐ecological model, young adults

## Abstract

**Background:**

Inadequate sleep and excess body weight are escalating global health concerns, especially among young adults facing academic pressures, digital lifestyles, and cultural norms. While quantitative research has shown links between sleep and body weight, little is known about the lived experiences shaping this relationship in Asian contexts.

**Methods:**

A qualitative study was conducted in urban Dhaka, Bangladesh, in 2022. Twenty‐six young adults aged 18–25 years were recruited through purposive and snowball sampling methods. Semi‐structured, in‐depth interviews were conducted in Bangla, audio‐recorded, transcribed verbatim, and translated into English. Thematic analysis was performed using an inductive approach, supplemented by deductive coding based on the socio‐ecological model (SEM).

**Results:**

Five key themes emerged: (1) structural and environmental conditions disrupting sleep; (2) digital night culture and socially reinforced wakefulness; (3) the prioritisation of productivity over sleep; (4) unequal burdens of household labour and caregiving and opportunities for rest; and (5) perceived associations between irregular sleep, fatigue, and eating behaviours. Young adults described poor sleep as both a personal and structural issue and perceived connections with body weight and overall wellbeing. Across the identified themes, sleep and eating practices that appeared individual were embedded within interpersonal, institutional, environmental, and sociocultural contexts.

**Conclusions:**

The findings indicate opportunities for healthy sleep are influenced by social, institutional, and environmental conditions in addition to individual practices. Consequently, sleep‐health strategies targeting young adults may benefit from integrating individual education with interventions that address peer and family practices, institutional expectations, and environments conducive to sleep health. Further longitudinal and mixed‐method research is required to investigate the pathways identified in these qualitative accounts.

**Patient and Public Contribution:**

The findings were discussed with patient and public involvement (PPI) members who are young adults. Their insights informed the development of the interview guide. A subset of participants helped interpret the results by engaging in member checking of preliminary findings and identified themes. Members of the public did not participate in study governance or manuscript preparation.

## 1 Introduction

In recent years, sleep disturbances have emerged as pressing public health challenges, particularly among young adults. Young adulthood is marked by evolving academic, occupational, social, and family responsibilities, as well as increased digital media use and irregular daily routines, all of which may influence sleep timing and quality [1, 2, 3]. Insufficient sleep is linked to numerous adverse health outcomes, such as cardiometabolic disorders and mental health conditions [4]. Previous studies relevant to obesity prevention have identified associations between short or poor‐quality sleep and appetite dysregulation, increased energy intake, reduced physical activity, and elevated body weight [5, 6, 7].

Obesity is a growing global public health challenge. Prevalence rates are rising rapidly among young people. The World Health Organisation (WHO) estimates overweight and obesity have nearly tripled since 1975. More than 340 million children and adolescents were classified as overweight or obese in 2016 [8, 9, 10]. Young people are particularly vulnerable because lifestyle habits formed during adolescence and early adulthood often persist throughout their lives [11, 12]. These habits influence long‐term health outcomes, including diabetes, cardiovascular disease, and mental health problems [13, 14, 15]. While dietary patterns, physical activity, and genetic factors are established determinants of obesity, emerging evidence highlights the important role of sleep quality in shaping body weight [1, 16]. Studies from high‐income countries (HICs) have demonstrated inadequate or poor‐quality sleep is associated with dysregulated appetite, increased caloric intake, reduced physical activity, and ultimately, a higher risk of obesity [5, 7]. Insufficient and poor‐quality sleep have been linked to cardiovascular disease, diabetes, mental health problems, and premature mortality [4]. Evidence suggests a bidirectional relationship between sleep disturbances and obesity, mediated by biological, behavioural, and psychosocial factors [6].

The majority of the research examining the relationship between sleep and weight has been epidemiological or clinically orientated, primarily focusing on quantifying associations among sleep duration, sleep quality, and body mass index [17]. While such evidence is valuable, it offers a limited perspective on the ways in which sleep practices are shaped within everyday contexts. Sleep is experienced within households, educational institutions, workplaces, neighbourhoods, and, increasingly, within digital social environments [18]. Therefore, the ability to achieve adequate sleep may be influenced not only by individual knowledge or motivation but also by interpersonal expectations, institutional requirements, physical environments, and broader social conditions [19]. Despite these findings, research on the sleep–obesity nexus remains limited in low‐ and middle‐income countries (LMICs), where rapid urbanisation, cultural practices, and social norms may uniquely shape both sleep behaviours and weight outcomes [20, 21, 22]. Factors such as overcrowded housing, environmental noise, heat, unreliable electricity, academic competition, evolving work patterns, and increased access to digital technologies can all affect the timing and quality of sleep among young adults [14, 23].

However, empirical evidence regarding the lived experiences of these influences remains limited, especially within South Asian contexts. While existing quantitative research can identify population‐level associations, it provides limited insight into how young adults interpret the relationships among sleep, fatigue, eating habits, social expectations, and perceived changes in body weight in daily life [24]. Young adulthood (18–25 years) is a critical period. During this time, individuals form long‐term health behaviours while managing intense academic, social, and economic pressures [22, 25, 26]. Worldwide, this age group faces irregular schedules, prolonged screen time, and heightened stress. These factors have been associated with disrupted sleep and weight‐related outcomes [11, 12, 27]. In LMICs, additional barriers, including overcrowded housing, erratic electricity, and cultural expectations, further limit young adults' ability to get adequate rest [14, 23].

Bangladesh represents a significant context for examining this issue. The country is undergoing rapid urbanisation, nutritional transition, and a rising prevalence of excess body weight, while undernutrition persists [28, 29, 30]. Evidence indicates a substantial burden of excess body weight among young people in Bangladesh, particularly in urban settings, while the role of sleep remains comparatively underexplored [21]. Urban young adults encounter academic pressures, pervasive digital technology, evolving food environments, and high‐density living conditions [31, 32]. These conditions may influence both sleep practices and weight‐related behaviours. However, the perspectives and experiences of young Bangladeshi adults regarding these relationships remain insufficiently explored.

The socio‐ecological model (SEM) provides a comprehensive framework for analysing this issue, as it conceptualises health behaviour as influenced by multiple levels rather than solely by individual choice. This study applied the SEM to examine how sleep‐related experiences are distributed across individual, interpersonal, institutional, and broader societal or environmental domains, consistent with the sleep‐focused ecological formulation described by Grandner and colleagues [25, 33]. At the individual level, relevant processes include sleep routines, fatigue, eating behaviours, and screen use. Interpersonal influences encompass peer communication, family expectations, and caregiving. Institutional influences comprise academic and workplace expectations, as well as the degree to which health services and universities recognise sleep as a health issue. Broader contextual influences consist of urban living conditions, infrastructure, cultural norms, and gendered expectations. Applying SEM in this manner enables the study to move beyond the identification of individual sleep behaviours and instead examine how seemingly personal practices are shaped, limited, or reinforced by interpersonal relationships and environmental contexts. This multilevel perspective is especially pertinent for understanding the potential gap between knowledge and practice. Young adults may acknowledge the significance of healthy sleep yet have limited opportunities to apply this knowledge when their sleeping environment, institutional obligations, or household responsibilities are beyond their control. The objective of this study was to investigate how young adults in urban Dhaka perceive and experience the interrelationships among sleep, eating behaviours, and body weight, and to examine how these experiences are situated across multiple levels of the Social Ecological Model.

## 2 Method

A qualitative study explored how young adults in urban Dhaka perceive and experience the relationships among sleep, eating behaviours, and body weight. A qualitative approach was appropriate as the study aimed to understand participants' experiences of sleep within their daily social, institutional, and environmental contexts, rather than to quantify associations or establish causality. The study design and analysis were guided by the SEM, which conceptualises health behaviours as shaped by multiple, interconnected levels of influence rather than by individual actions alone. This framework was applied deductively to identify influences at various ecological levels and used alongside inductive coding to allow new patterns to emerge from participants' accounts.

### 2.1 Study Setting and Participants

The study was conducted in urban Dhaka, Bangladesh. Dhaka represents a significant context for investigating young adults' sleep experiences, as daily routines unfold within a rapidly urbanising environment marked by high population density, increasing digital connectivity, and evolving educational, occupational, and social practices. Participants were eligible if they were aged 18–25 years, lived in Dhaka, and were willing to discuss their experiences of sleep, lifestyle behaviours, and body weight. Participants were recruited through purposive and snowball sampling methods. Purposive recruitment sought variation in gender, educational background and socioeconomic circumstances, supplemented by snowball sampling; however, the achieved sample was predominantly unmarried and student based. This sample composition is considered when assessing the transferability of the study's findings. A total of 26 young adults participated.

### 2.2 Data Collection

In 2022, semi‐structured, in‐depth individual interviews (IDIs) were conducted. The interview guide was developed based on the SEM and examined participants' sleep routines, eating behaviours, interpersonal relationships, family and peer expectations, academic or occupational demands, living environments, digital practices, and broader sociocultural influences. This approach enabled participants to articulate their individual experiences as well as the contextual factors that influenced them. The interview guide was initially developed in English and subsequently translated into Bangla. The guide underwent review for cultural and linguistic appropriateness and was refined based on feedback from pilot testing. Interviews were conducted in Bangla according to participants' preferences, lasted approximately 45–65 min, and were audio‐recorded with informed consent. Field notes documented relevant contextual observations. Sample adequacy was evaluated based on the richness and relevance of the interview data in addressing the study's specific aim. The final sample of 26 participants produced detailed accounts across the principal areas of inquiry and yielded sufficient information to facilitate the development and refinement of themes. The study does not claim statistical representativeness. Transferability should be evaluated with consideration of the sample's characteristics and the specific research context. Interviews were conducted in Bangla and subsequently translated into English for analysis and reporting. To minimise loss of meaning across languages, translations were checked against the Bangla transcripts and audio recordings by a bilingual researcher. Additionally, culturally specific expressions were discussed within the research team. However, some culturally specific meanings may have been attenuated during translation, which is acknowledged as a limitation of the study.

### 2.3 Data Analysis

Interviews were transcribed verbatim and translated into English. The analysis followed Braun and Clarke's [34] approach to thematic analysis. The data were initially reviewed through repeated reading of transcripts, re‐listening to interview recordings, and examination of field notes. Significant statements were identified, and preliminary codes were developed through an iterative process. The coding process incorporated both inductive and deductive approaches. Inductive coding allowed patterns to emerge from participants' accounts without restriction to predetermined theoretical categories. In contrast, deductive coding was guided by the SEM, which sensitised the analysis to influences across ecological levels. Codes were compared, grouped into broader categories, and progressively refined into candidate themes. Two researchers coded the interview data, after which the broader research team reviewed and discussed the coding decisions and theme development. Themes were subsequently reviewed against coded extracts and the wider dataset, refined through research team discussions, and examined in relation to the SEM. The SEM was not used solely to assign each theme to a single ecological level.

Instead, the SEM facilitated examination of how themes spanned multiple levels. For example, individual behaviours such as late‐night screen use or fatigue‐related eating were considered within the context of peer relationships, institutional expectations, household responsibilities, and material living conditions. This cross‐level interpretation informed the development of the final thematic structure. Table S1 presents an illustrative coding framework showing the relationships among selected codes/subthemes, final themes, supporting participant accounts, and their positioning within the SEM. Several strategies were employed to enhance analytic rigour and trustworthiness. These strategies included repeated engagement with interview material, maintenance of an audit trail documenting coding and theme development, peer debriefing within the research team, and member checking with a subset of participants. Preliminary findings and themes were presented to four participants for member checking. Participants assessed whether the emerging interpretations accurately represented their experiences and highlighted any aspects needing clarification or further refinement. Participants' feedback was incorporated into the final theme development. Reflexive discussions within the research team were conducted to consider alternative interpretations and to assess the influence of researcher assumptions during theme development.

## 3 Results

There were 26 IDIs conducted with young Bangladeshi adults. Participants had a mean age of 21.85 years (SD = 1.35; range 20–25 years). The key characteristics of participants are summarised in Table 1.

**Table 1:** Characteristics of participants (*N* = 26).

		*N* (%)
Gender	Male	12 (46.2)
	Female	14 (53.8)
Level of education	Higher secondary	1 (3.8)
	Undergraduate	20 (76.9)
	Postgraduate	5 (19.2)
Occupation	Student	24 (92.3)
	Employed	2 (7.7)
Type of family	Nuclear	20 (76.9)
	Extended	6 (23.1)
Ethnicity	Bengali	26 (100.0)
Marital status	Unmarried	25 (96.2)
	Married	1 (3.8)
Monthly family income (BDT)	≤ 10,000	4 (15.4)
	10,001–40,000	12 (46.2)
	> 40,000	10 (38.5)
Living arrangement	Hostel	19 (73.1)
	Home	7 (26.9)
BMI Category	Underweight	1 (3.8)
	Normal weight	13 (50.0)
	Overweight	10 (38.5)
	Obese	2 (7.7)

The analysis identified five interconnected themes that spanned multiple levels of the SEM: (1) structural and environmental conditions disrupting sleep; (2) digital night culture and socially reinforced wakefulness; (3) the prioritisation of productivity over sleep; (4) unequal burdens of household labour and caregiving and opportunities for rest; and (5) perceived associations between irregular sleep, fatigue, and eating behaviours. Table 2 summarises the ecological positioning of these themes.

**Table 2:** Ecological positioning of themes and illustrative influences within the SEM.

Theme	Individual	Interpersonal	Institutional	Broader societal/environmental
Structural and environmental conditions disrupting sleep	Stress, fatigue	Shared sleeping environments	—	Heat, electricity disruption, noise, overcrowding, financial/material constraints
Digital night culture and socially reinforced wakefulness	Screen use, delayed sleep	Peer messaging, gaming, late‐night socialising	—	Digitally mediated social norms
Prioritisation of productivity over sleep	Sleep sacrifice, stress	Family expectations	Academic/workplace expectations; limited sleep‐health recognition	Productivity norms
Unequal household labour, caregiving and opportunities for rest	Fatigue, reduced rest	Household labour and caregiving	—	Gendered expectations concerning domestic responsibilities and rest
Perceived links between irregular sleep, fatigue and eating	Fatigue‐related eating, snacking, appetite experiences	Peer eating/socialising	Academic schedules	Late‐night food availability/urban food environment

### 3.1 Theme 1: Structural and Environmental Conditions Disrupting Sleep

Participants experienced challenges in obtaining adequate sleep as a result of material and environmental conditions outside their immediate control. Factors such as financial insecurity, excessive heat and humidity, electricity interruptions, noise, overcrowded living arrangements, and restricted control over sleeping environments were identified as important barriers to restful sleep. These findings suggest sleep should be understood not solely as an individual behaviour but as a practice influenced by access to material and environmental resources. Financial concerns persisted into the night, manifesting as worries about family expenses, tuition fees, employment prospects, and future responsibilities. Participants associated these ongoing concerns with difficulty initiating sleep and subsequent fatigue. Participants linked fatigue to increased reliance on inexpensive or convenient foods, which they perceived as contributing to changes in their weight.

Honestly, money is always on my mind. My family struggles with expenses, and I feel the pressure to support them after graduation. That stress keeps me awake at night. Even when I lie down, I keep thinking about bills, fees, and job uncertainty. Because of that, I sleep very late, and the next day I feel weak and tired. To cope, I eat whatever is available, often cheap fast food, because it's quick and fills me up. But deep down I know it's not healthy, and it's slowly affecting my weight.

(IDI02, Male)

Environmental and infrastructural conditions were also identified as important influences on sleep patterns among participants. Participants described experiencing high summer temperatures and humidity in conjunction with electricity interruptions or load‐shedding, especially in the absence of fans or other cooling methods during outages. Participants described these conditions as making restful sleep difficult during certain periods and associated them with restless and uncomfortable nights and repeated awakenings. Participants experienced frequent awakenings due to excessive sweating and inadequate ventilation. Instead of attributing weight gain directly to these conditions, participants associated disrupted sleep and subsequent fatigue with increased consumption of heavier, fried, or convenient foods.

The weather here makes sleeping so hard. During the summer months, it's so humid and sticky that I can't fall asleep, even with the fan on full speed. If there's load‐shedding, the whole night feels unbearable. I keep waking up, sweating, and just waiting for morning. The next day, because I am exhausted, I end up eating heavy meals or fried food to get some energy. Over time, this lack of proper sleep and overeating makes me feel my body is changing; I'm gaining weight, but I don't feel strong or healthy.

(IDI05, Male)

Participants emphasised that their living environments had an impact on their ability to sleep. Participants described residing in densely populated urban areas. Noise pollution from traffic, rickshaws, and street activity was pervasive. Overcrowding within homes further exacerbated these challenges. Sharing small rooms with siblings often resulted in disruptions from late‐night studying or phone use. This reduced opportunities for uninterrupted rest. Additional environmental irritants, such as mosquitoes, further fragmented sleep. Participants associated disrupted sleep with next‐day fatigue and changes in eating, including increased consumption of rice and snacks to maintain energy. Participants reported waking up fatigued and craving additional food, particularly rice or snacks, to maintain energy throughout the day. Participants described recurring associations between disrupted sleep, altered eating and perceived changes in body weight.

Where we live, it's always noisy: rickshaws, traffic, and people shouting in the streets. Our house is small, and I share a room with my siblings. If one of us studies late at night or uses the phone, the others can't sleep properly. Sometimes mosquitoes make it worse; I wake up multiple times just to swat them away. All of this means my sleep is broken. And when I don't sleep well, I crave more food the next day. I end up eating extra rice or snacks, just to keep going. Slowly, this is affecting my health and weight.

(IDI07, Female)

### 3.2 Theme 2: Digital Night Culture and Socially Reinforced Wakefulness

Participants highlighted how sleep behaviours were shaped by social and cultural expectations. Night‐time gatherings with peers included food and drinks that participants associated with weight‐related challenges. Participants felt compelled to adjust their routines to meet social expectations or maintain relationships, which disrupted sleep and influenced weight‐related behaviours. As one participant reflected:

In Dhaka, it's common for young people like us to hang out late at tea stalls or cafes. We stay up until midnight or later, chatting, eating parathas, and drinking tea. It's fun, but the next morning we feel lazy, and this habit definitely makes it harder to control weight.

(IDI09, Male)

Digital media were integral to participants' night‐time social experiences. Participants reported sustained engagement with streaming platforms, social media, messaging, gaming, and other screen‐based activities that participants described as prolonging wakefulness beyond intended bedtimes. Notably, these behaviours were not always perceived as individual choices. Digital communication facilitated peer interaction and fostered expectations of ongoing social availability during late‐night hours. Participants reported habitual late‐night smartphone use and media streaming. Interpersonal influences, including peer communication and participation in online gaming communities, further reinforced these sleep‐disrupting behaviours.

Scrolling on my phone at night is almost a ritual. I know it cuts into my sleep, but I lose track of time. The lack of sleep makes me too tired to exercise the next day, and I rely on sugary foods to get through. Slowly, I've realised it's not just food choices, but sleep that's affecting my body shape.

(IDI22, Female)

My friends always call me late at night to chat or study together. Even though I know it disrupts my sleep, I feel I can't say no. The next day, I feel sleepy and tend to snack more.

(IDI04, Male)

A global digital night culture, characterised by technology‐facilitated social interactions and extended leisure time, was identified as undermining circadian rhythms and healthy lifestyle practices. Participants' accounts indicated digital wakefulness functions at both individual and interpersonal levels. While individuals may choose to continue watching, scrolling, messaging, or gaming, these activities often take place within digitally mediated peer relationships, where maintaining connectivity becomes integrated into typical night‐time social life.

Even when I finish studying, I lie in bed scrolling TikTok. I know it destroys my mornings, but it feels impossible to stop it. It's like my whole friend group lives online after midnight.

(IDI19, Male)

Netflix is the killer. I keep telling myself, ‘Just one more episode,’ until suddenly it's 3 a.m. The next day, I skipped breakfast and snacked on junk food because I'm too tired to cook.

(IDI12, Female)

These accounts conceptualise digital night culture as a collective phenomenon rather than solely an individual screen‐use behaviour. Night‐time wakefulness is integrated into entertainment routines and peer interactions, and participants attribute the resulting fatigue to disrupted meals and increased dependence on convenient foods.

### 3.3 Theme 3: The Prioritisation of Productivity Over Sleep

Participants characterised sleep as a resource that could be reduced or postponed when academic, occupational, or family demands required additional time. Academic pressure, examinations, concerns about future employment, and expectations of achievement were identified as factors that deprioritised rest. Sleep sacrifice was not merely attributed to poor time management; rather, participants' accounts indicated that reduced sleep could become normalised as an aspect of productivity or commitment. One participant described:

As a university student, I'm always stressed about exams and family expectations. Even when I go to bed on time, my mind keeps racing. Poor sleep makes me crave sweet foods, and I tend to eat more than I should. I feel like stress, sleep, and weight are all linked together in my life.

(IDI02, Male)

Sleep was described as the first commodity to be sacrificed when navigating the demands of academics and work by participants. Participants normalised reduced sleep as a necessary part of achieving their goals, while at the institutional level, participants perceived educational and workplace expectations as reinforcing this pattern.

My whole schedule is upside down: late‐night classes, online shows, social media. Sleep is the last priority. During exams, I cut my sleep down to three or four hours. Professors advise us to manage our time, but the system often rewards those who stay up all night. It feels like sleeping is a luxury only the lazy can afford.

(IDI17, Male)

At my company, staying late is praised as being committed. Even when we're exhausted, nobody talks about rest; we just push through. (IDI18, Male)

Participants described limited institutional recognition and support for sleep health within universities, healthcare settings, families and workplaces. They also emphasised that sleep remains largely absent from discussions on health promotion and policy.

Our university runs workshops on diet and exercise, but never on sleep. It's treated like something you should figure out yourself, even though we know it affects our health.

(IDI09, Male)

Participants expressed concern about the lack of recognition of sleep as a legitimate health issue within the healthcare system, families, and educational institutions. Attention remained focused on visible or acute illnesses. Participants described sleep concerns as being trivialised or dismissed as stress or overthinking and perceived sleep as receiving limited attention within broader health‐promotion and disease‐prevention strategies. They described managing sleep difficulties largely on their own, sometimes through coping practices such as overeating or consuming readily available foods. Participants situated these coping behaviours within a broader perceived lack of institutional recognition and support for sleep health.

In our area, no one talks about sleep as a health issue. Doctors don't ask, families don't care, everyone just focuses on physical illness. If I complain about poor sleep, people say, ‘It's just stress, don't overthink.’ But this stress and sleeplessness push me toward overeating and junk food, which is slowly changing my body. I believe there is a lack of proper support systems for young people like us, whether from universities, workplaces, or healthcare providers. We are just managing on our own.

(IDI23, Male)

Participants described a dual institutional message in which sleep was portrayed as expendable in favour of academic productivity, yet it received minimal emphasis in university health promotion initiatives. This perceived institutional neglect further reinforced the notion that sleep is an individual responsibility rather than an aspect of health that warrants organisational support.

### 3.4 Theme 4: Unequal Burdens of Household Labour, Caregiving and Opportunities for Rest

Female participants reported that household labour and caregiving responsibilities diminished the time available for rest. Their accounts described cooking, cleaning and caring for younger family members after study or work commitments, with personal study and sleep postponed until household responsibilities were fulfilled. One participant explicitly contrasted her routine with that of her brothers:

After classes, my brothers rest, but I help with cooking and cleaning. Only when everyone is asleep can I open my books. That means my day often stretches until 2 to 3 a.m., and the next morning I'm too tired to eat healthy or exercise.

(IDI15, Female)

A second participant similarly described responsibility for younger siblings:

Even though I'm only 22, I take care of my younger siblings after work. The men in my house don't share this. By the time I go to bed, it's late, and I wake up hungry, eating whatever is quick.

(IDI22, Female)

These accounts indicated that opportunities for rest were not equally distributed within certain households. Sleep time often competed with gendered domestic and caregiving responsibilities, rendering access to rest a relational issue rather than an exclusively individual one. These experiences connected interpersonal household practices to broader gender expectations. Therefore, this finding should be interpreted as evidence of gendered differences in access to rest as reported by participants, rather than as evidence that women universally experience poorer sleep than men.

### 3.5 Theme 5: Perceived Links Between Irregular Sleep, Fatigue, and Eating Behaviours

Participants described perceived connections between irregular sleep, fatigue and changes in eating behaviour. Late‐night studying and extended wakefulness were associated in their accounts with fatigue, altered hunger, convenience‐oriented food choices and perceived weight change. These accounts represent participants' interpretations and do not establish the direction or physiological basis of these relationships. One participant explained:

Because of my late‐night study sessions, I usually sleep after 2 a.m. I wake up feeling tired and hungry, so I grab whatever food is quick, usually fried snacks or instant noodles. Over time, I noticed my weight going up, but I never thought sleep could be connected.

(IDI05, Male)

The account associated delayed sleep with subsequent fatigue, increased hunger, convenience‐oriented food choices, and perceived weight change. However, the participant's description does not clarify the causal direction of these relationships. Participants' accounts described perceived behavioural connections between disrupted sleep, fatigue and subsequent eating practices. Participants described eating not from genuine hunger but as a coping strategy for fatigue:

When I don't sleep well, I feel restless the next day. I keep eating just to stay awake in class or at work. It's not real hunger, but I use food as a way to fight off sleepiness. My weight has slowly increased this way.

(IDI07, Female)

Participants reported using eating as a means to manage tiredness rather than to satisfy hunger. In these narratives, food served as a coping mechanism for fatigue.

When I'm stressed about exams, I can't sleep properly. I end up eating sweets at night to calm myself, and my weight has increased over the last few months.

(IDI15, Female)

Late‐night tea stalls and street food shops are everywhere. When I can't sleep, I often go out to eat with friends, which definitely affects my weight.

(IDI23, Male)

These eating practices were not solely individual in nature. Participants identified examination stress, peer socialising, and the availability of late‐night food outlets as contexts in which sleep and eating behaviours became interconnected. Consequently, this theme spanned multiple ecological levels, connecting individual experiences of fatigue and appetite with academic demands, peer practices, and the urban food environment. The five themes indicated participants did not experience sleep as isolated individual behaviour. Opportunities for rest were shaped by material living conditions, digital and peer practices, institutional expectations, household responsibilities, and broader gendered and urban contexts. Participants also identified connections between disrupted sleep, fatigue, and eating behaviours, with these accounts situated within the same multilevel conditions. SEM thus demonstrated that behaviours often attributed to individual choice occurred within broader social and structural opportunities and constraints.

## 4 Discussion

This study explored the perceptions and experiences of young adults in urban Dhaka regarding the interrelationships among sleep, eating behaviours, and body weight within their daily social and environmental contexts. The central finding was that participants not only associated inadequate sleep with eating behaviours and perceived weight changes but also recognised that opportunities for healthy sleep were influenced by factors operating across multiple ecological levels. Participants identified financial concerns, heat, electricity disruptions, noise, and crowded living conditions, as well as digitally mediated social interactions, academic and productivity expectations, and household and caregiving responsibilities. They further linked disrupted or irregular sleep to fatigue, increased consumption of convenient or energy‐dense foods, snacking, and perceived changes in body weight. A key finding was that sleep was not perceived solely as a behaviour governed by individual control. Participants identified noise, crowded and shared sleeping environments, heat, humidity, mosquitoes, electricity interruptions, and financial concerns as important barriers to achieving uninterrupted rest.

Participant accounts were especially compelling in detailing how traffic and street noise, room‐sharing, load‐shedding, and heat disrupted their ability to sleep, even when they intended to rest. This finding aligns with existing literature that identifies housing and environmental conditions as important determinants of sleep quality in urban populations [35, 36]. These experiences occur within Bangladesh's broader urban and nutritional transition, characterised by changing food environments, reduced physical activity and an increasing burden of excess body weight [14, 37]. This study explored how participants perceived urban and sociocultural conditions as shaping their sleep practices and weight‐related behaviours. Participants described noise, congestion and constrained residential environments as barriers to restful sleep and perceived subsequent fatigue as influencing their eating. These experiences are consistent with the global literature linking poor sleep to obesity and metabolic risk; however, the narratives illustrate how these concerns are experienced within the rapidly urbanising context of Dhaka [38]. Participants' accounts suggest that inadequate sleep may become normalised within demanding urban routines, particularly where environmental barriers coexist with limited institutional attention to sleep health [39].

The timing of data collection warrants careful consideration. Interviews took place in 2022, a period marked by pressures on Bangladesh's electricity supply and substantial disruptions to higher education and students' daily lives due to the COVID‐19 pandemic [40, 41]. These circumstances are particularly relevant to participants' reports of electricity disruption, heat, and sleep difficulties, and may have increased the prominence of these experiences during data collection. More broadly, the power sector in Bangladesh continues to encounter challenges related to energy security, supply‐demand pressures, and the provision of reliable electricity [42]. In contrast, participants' accounts of dense urban living, environmental noise, shared sleeping environments, and financial pressures may reflect more persistent constraints. Therefore, these findings should be interpreted as historically and contextually specific, rather than representative of all young adults in Bangladesh.

Young adults are facing more stressors that can impact their sleep quality. Previous research from HIC has noted the role of social networking in disrupting adolescent sleep. Young adults are at risk for unique health problems [20, 26]. In LMIC contexts, late‐night screen use has increased due to rapid urbanisation and the widespread use of low‐cost mobile devices, frequently without corresponding public awareness of healthy sleep practices [43, 44]. The present findings indicate an exclusive focus on ‘screen time’ may overlook the interpersonal functions of digital engagement. Participants did not remain awake solely due to device availability; rather, digital platforms facilitated social connection, entertainment, academic activities, and expectations of availability. Consequently, wakefulness may be reinforced through social mechanisms. This distinction demonstrates the analytical utility of SEM. An individual‐level interpretation may identify excessive screen use as the primary issue and recommend reducing exposure before bedtime. In contrast, a multilevel interpretation examines the underlying reasons for persistent screen use and the interpersonal expectations or social routines that sustain it. Therefore, sleep‐health strategies targeting young adults may be more effective if they address peer norms and digitally mediated social practices in addition to individual screen‐management skills.

Academic pressure and expectations regarding achievement constituted an important context influencing participants' sleep patterns [45]. Participants identified examinations, study schedules, family expectations, and concerns about future employment as primary factors contributing to the postponement or reduction of sleep. Notably, sleep was described as ‘a luxury only the lazy can afford,’ highlighting how insufficient sleep may be perceived as a marker of commitment and productivity, rather than merely a result of poor time management [46]. Previous research has identified academic workload, irregular schedules, and stress as important correlates of inadequate sleep among students and young adults [11, 47]. Competitive tests for admission and a lack of job opportunities frequently increase academic pressure, resulting in long‐term stress and unstable study habits [47, 48, 49]. The present findings suggest institutional and cultural expectations may contribute to the normalisation of sleep sacrifice. Participants simultaneously encountered messages about managing their own time while perceiving academic environments as rewarding extended study and giving relatively little attention to sleep within health‐promotion activities. This apparent contradiction is important. It places responsibility for healthy sleep on the individual while potentially maintaining institutional conditions in which rest is deprioritised.

Participants' accounts therefore support consideration of sleep not only within individual health education but also within university wellbeing, academic scheduling and student‐support systems. Female participants' narratives revealed an additional dimension: access to rest was influenced by household labour and caregiving responsibilities. Participants reported completing cooking, cleaning, or caregiving tasks after academic or work commitments, often postponing their own study and sleep until these household duties were fulfilled. One participant explicitly contrasted her responsibilities with her brothers' opportunities for rest, while another noted that male household members did not share caregiving responsibilities. These accounts align with existing literature indicating gendered household roles can limit women's opportunities for leisure, physical activity, and sleep [23, 50]. Participants connected irregular or insufficient sleep with changes in eating behaviour. Their accounts included eating to stay awake, choosing fried or convenient foods when fatigued, eating sweets during periods of examination stress, skipping breakfast after late‐night wakefulness, and eating socially at late‐night food outlets. Participants also perceived that these patterns were accompanied by increases in their body weight.

These perceptions align with quantitative and physiological research that links inadequate sleep to disruptions in appetite regulation, increased food intake, and higher body weight [5, 51, 52]. Previous research indicates poor sleep can lead to hormonal changes that affect appetite and metabolism, potentially creating a cycle of weight gain and worsening sleep issues. Stress‐induced sleep disruption is a recognised predictor of obesity [5, 51, 52]. Poor sleep quality is associated with dysregulated appetite, increased snacking, and weight gain. Participants reported associations between late‐night wakefulness, fatigue, and increased consumption of convenient or energy‐dense foods. These experiences were situated within academic schedules, peer influences, and the accessibility of late‐night food options. As the sample primarily consisted of students, with only two employed individuals, workplace‐related accounts should be regarded as illustrative rather than representative of employed young adults. These findings indicate that incorporating sleep considerations may enhance nutritional interventions by highlighting the social and temporal contexts in which eating behaviours occur.

Participants demonstrated awareness that late‐night screen use, insufficient sleep, or irregular routines were undesirable. However, possessing this knowledge did not necessarily enable them to alter their circumstances. Factors such as environmental noise, heat during electricity interruptions, shared rooms, academic demands, peer expectations and household responsibilities restricted participants' opportunities for healthier sleep. Participants also described limited institutional recognition of sleep, perceiving that healthcare providers, families and universities did not consistently treat sleep as a legitimate health concern. These findings indicate the issue is more accurately conceptualised as a knowledge–opportunity gap rather than merely a knowledge–practice gap. This distinction is important because attributing unsuccessful behaviour change solely to knowledge, motivation, or self‐efficacy risks individualising circumstances that are structurally constrained. Awareness of the benefits of adequate sleep does not eliminate environmental noise, restore electricity during load‐shedding, redistribute household caregiving, or independently alter institutional expectations regarding productivity. This interpretation links the environmental and behavioural findings, thereby providing a stronger foundation for multilevel interventions compared to sleep education alone.

The SEM provided analytical value by illustrating how health behaviours that might initially appear individual were embedded within influences spanning multiple ecological levels in participants' accounts. For example, late‐night screen use reflected individual choices but was reinforced by peer communication and prevailing digital social norms. Decisions to sacrifice sleep for study were shaped not only by personal priorities but also by family expectations and institutional productivity standards. Fatigue‐related eating was influenced by academic schedules, peer social interactions, and the accessibility of convenient late‐night foods. Additionally, opportunities for rest within households were influenced by interpersonal responsibilities and broader gender‐based expectations. The significance of this multilevel interpretation lies not only in categorising determinants within distinct SEM levels, but also in demonstrating the interconnectedness of influences across these levels in participants' experiences. This perspective challenges explanations that attribute inadequate sleep primarily to individual sleep hygiene and indicates that strategies focused solely on individual behaviour change may be insufficient if interpersonal, institutional, and environmental constraints persist.

### 4.1 Strengths and Limitations

A principal strength of this study lies in its qualitative exploration of young adults' understandings of sleep, eating, and body weight concerns within the social and material contexts of daily life in urban Bangladesh. The thematic analysis incorporated inductive coding and SEM‐informed deductive coding. Additionally, in‐depth interviews provided contextual insights that are not readily accessible through conventional quantitative measures such as sleep duration or body mass index.

Several limitations warrant consideration. First, the findings reflect participants' perceptions and experiences and do not establish causal or temporal relationships among sleep, eating behaviours, and body weight. Sleep, dietary intake, and longitudinal weight change were not objectively measured; thus, participants' accounts of weight change and sleep‐related eating should be interpreted as perceived associations rather than verified physiological effects. Second, the final sample was predominantly unmarried and consisted mainly of students from urban Dhaka. As a result, transferability to employed, out‐of‐school, rural, and more socioeconomically diverse young adults is limited. Recruitment through institutional networks and subsequent snowball sampling may also have favoured participants with similar educational and social backgrounds.

Third, interviews were conducted in 2022. Period‐specific factors, such as electricity disruptions and post‐pandemic changes in educational and social routines, may have heightened the prominence of certain reported sleep barriers. Fourth, interviews were conducted in Bangla and translated into English. Cross‐language qualitative analysis may attenuate culturally specific meanings, and this limitation should be acknowledged. Fifth, gender‐related interpretations were primarily informed by women's accounts. While the findings highlight perceived gendered differences in access to rest, they do not establish the comparative prevalence of sleep difficulties between women and men. Finally, the limited representation of employed young adults constrains conclusions regarding workplace cultures; workplace‐related accounts should therefore be interpreted with caution. Further longitudinal and mixed‐method research is required to test the pathways suggested by these qualitative accounts and to determine the applicability of these findings to diverse groups of young adults in Bangladesh.

## 5 Conclusion

This study offers insight into the ways young adults in urban Dhaka perceive and experience the relationships among sleep, eating behaviours, and body weight within their daily social and environmental contexts. Participants identified opportunities for healthy sleep as influenced by material living conditions, digitally mediated social interactions, academic and institutional demands, as well as household and caregiving responsibilities. Additionally, participants noted perceived links between disrupted sleep, fatigue, eating practices, and changes in body weight; however, these qualitative accounts do not establish causal or physiological pathways. When analysed using SEM, the findings suggest that sleep should not be understood solely as an individual issue related to knowledge, motivation, or sleep hygiene. Instead, participants' accounts highlight a knowledge–opportunity gap, where awareness of healthy sleep does not necessarily lead to practice if social, institutional, and environmental factors limit opportunities for rest. Consequently, sleep‐health strategies for young adults may benefit from supplementing individual education with approaches addressing peer and family practices, institutional expectations, gendered responsibilities and environments conducive to sleep.

### 5.1 Policy and Practice Recommendations

The current findings indicate that sleep health should be integrated with nutrition and physical activity in health‐promotion initiatives targeting young adults. At the individual level, sleep education can enhance understanding of healthy sleep practices and the interrelationships among sleep, fatigue, and eating behaviours. Nevertheless, the knowledge–opportunity gap identified in participants' accounts suggests that education alone is inadequate when opportunities for healthy sleep are limited.

At the interpersonal level, incorporating peer and digitally mediated social practices into sleep‐health initiatives is recommended, rather than attributing night‐time screen use solely to individual self‐control. Family‐oriented strategies should also acknowledge that household responsibilities and expectations shape opportunities for rest. Furthermore, gendered accounts indicate that sleep health intersects with the distribution of unpaid domestic and caregiving labour. Gender‐responsive health promotion may therefore be enhanced by encouraging a more equitable division of household responsibilities.

At the institutional level, universities are encouraged to integrate sleep into student wellbeing and health‐promotion programs, alongside nutrition, physical activity, and mental health. Participants' observations that sleep is largely absent from institutional health messaging support the inclusion of brief sleep‐health assessments within university health or counselling services. Additionally, academic institutions should evaluate how scheduling, workload expectations, and productivity cultures influence students' opportunities for adequate rest. The environmental findings expand the scope of sleep health beyond traditional health services. Participants' reports of noise, overcrowding, heat, and inadequate ventilation indicate that sleep should be addressed within urban and housing policy. Interventions such as noise management, improved ventilation in student accommodation, and sleep‐supportive residential design merit consideration as elements of a comprehensive public health strategy. These recommendations are based on participants' experiences; their effectiveness was not assessed in this study and warrants further investigation.

### 5.2 Patient or Public Contributions

Young adults participated in refining the interview guide during pilot testing to improve its cultural and linguistic relevance. Additionally, a subset of participants engaged in the interpretive process by member checking preliminary findings. Members of the public did not participate in study governance or manuscript preparation.

### 5.3 Scope

This study explored how structural, social, and behavioural factors shape young adults' experiences of sleep, eating, and perceived body‐weight change in urban Bangladesh. By centring young adults' lived experiences and incorporating participant feedback through member checking, this study provides evidence relevant to culturally responsive, youth‐focused sleep health promotion. The findings also show the importance of involving young adults in interpreting health research on behaviours within their daily social and environmental contexts.

## Author Contributions

**Mst Sabrina Moonajilin:** conceptualisation, methodology, writing – original draft. **Rajib Ul Islam:** data curation, writing – review and editing. **Labina Taher:** data curation; writing – review and editing. **Fariha Noshin:** data curation, writing – review and editing. **Tabeen Taher:** data curation, writing – review and editing. **Sabkat Kamal:** data curation, writing – review and editing.

## Funding

The authors have nothing to report.

## Ethics Statement

Ethics approval and consent to participate. All procedures performed in studies involving human participants were in accordance with the ethical standards of the institutional and/or national research committee and with the 1964 Helsinki Declaration and its later amendments or comparable ethical standards. The ethical aspects of this study were also reviewed and approved by the Biosafety, Biosecurity, and Ethical Review Board of Jahangirnagar University, Savar, Dhaka‐1342, Bangladesh [Ref No: BBEC, JU/M 2022/19 (6)]. All responses were anonymous, ensuring data confidentiality. All participants provided informed consent after being informed about the purpose of the study. Consent for publication: All authors.

## Conflicts of Interest

The authors declare no conflicts of interest.

## Supporting information


Supporting File

## Data Availability

The data that support the findings of this study are available on request from the corresponding author. The data are not publicly available due to privacy or ethical restrictions.
