# Supplementary material for: Losing Sleep, Gaining Weight? Young Bangladeshi Adults' Perceptions of Sleep, Eating and Body Weight: A Qualitative Study
Source: Health Expect. 2026 Oct 16;29(5):e70933. doi: 10.1111/hex.70933 (PMC13674411; doi:10.1111/hex.70933)
Supplement: Supplementary file 1 [file HEX-29-e70933-s001.docx]

Supplementary Table

S1. *Illustrative coding framework*

| **Final theme** | **Illustrative codes/subthemes** | **Illustrative data supporting coding** | **SEM level(s)** |
| --- | --- | --- | --- |
| 1. Structural and environmental conditions disrupting sleep | Financial stress and insecurity; worries about tuition/family expenses/future employment; heat and humidity; electricity interruption/load-shedding; inadequate ventilation; traffic and street noise; overcrowded/shared sleeping environments; mosquitoes/environmental disturbance | “Money is always on my mind… bills, fees, and job uncertainty… I sleep very late…” (IDI02, male); “If there’s load-shedding, the whole night feels unbearable…” (IDI05, male); “Our house is small, and I share a room with my siblings…” (IDI07, female) | Individual; interpersonal; broader societal/environmental |
| 2. Digital night culture and socially reinforced wakefulness | Ritualised late-night phone scrolling; streaming beyond intended bedtime; online gaming; peer messaging; expectations of late-night social availability; peer-based online engagement; late-night tea-stall/café socialising | “Scrolling on my phone at night is almost a ritual…*” (IDI22, Female)*; “It’s like my whole friend group lives online after midnight” (IDI19, male); “Netflix is the killer… ‘Just one more episode’…” (IDI12, female); late-night tea-stall/café socialising (IDI09, male) | Individual; interpersonal; broader societal/environmental |
| 3. The prioritisation of productivity over sleep | Academic stress and deadlines; late-night studying; sleep sacrifice for examination performance; normalisation of insufficient sleep; productivity expectations; work culture valorising staying late; limited institutional recognition of sleep health | “Our whole life is centred around exams and results…” (IDI17, male); “At my company, staying late is praised as being committed… nobody talks about rest; we just push through.” (IDI18, male) | Individual; interpersonal; institutional; broader societal/environmental |
| 4. Unequal burdens of household labour, caregiving and opportunities for rest | Domestic responsibilities extending waking hours; cooking and cleaning after study; caregiving for younger siblings; unequal distribution of household labour; postponement of personal study/sleep; gendered expectations concerning rest | “After classes, my brothers rest, but I help with cooking and cleaning…” (IDI15, female); “I take care of my younger siblings after work. The men in my house don’t share this…” (IDI22, female) | Individual; interpersonal; broader societal/environmental |
| 5. Perceived links between irregular sleep, fatigue and eating behaviours | Fatigue-related eating; eating to remain awake; perceived changes in hunger/appetite; convenience-oriented food choices; fried foods/instant noodles; sweets during examination stress; late-night eating with peers; perceived weight change | “I wake up feeling tired and hungry, so I grab whatever food is quick…” (IDI05, male); “I keep eating just to stay awake…” (IDI07, female); “I end up eating sweets at night to calm myself…” (IDI15, female); “Late-night tea stalls and street food shops are everywhere…” (IDI23, male) | Individual; interpersonal; institutional; broader societal/environmental |
